# The accuracy and safety of intraoperative ultrasound-guided external ventricular drainage in intraventricular hemorrhage

**DOI:** 10.1038/s41598-023-38567-y

**Published:** 2023-07-17

**Authors:** Lijun Zhang, Zhaohui Mu, Guoliang Shen, Ming Yang

**Affiliations:** 1 Department of Neurosurgery, Taizhou First People’s Hospital, Taizhou, Zhejiang 318020 People’s Republic of China; 2 Department of Neurosurgery, Wenzhou Medical University Affiliated Huangyan Hospital, Taizhou, Zhejiang 318020 People’s Republic of China

**Keywords:** Neuroscience, Neurology

## Abstract

Severe IVH often results in a poor outcome. Currently, EVD is a standard treatment for IVH, but there is little research to show whether using ultrasound to guide the catheter placement improves outcome. Patients with severe IVH who had iUS-guided EVD (the iUS-guided group) were enrolled retrospectively and compared with a group who had EVD performed without ultrasound guidance (the control group) from January 2016 to July 2022. Data were collected on accuracy of the catheter placement, complications and outcome at 3 months assessed by mRS. The accuracy of the EVD placement was classified as optimal placement, sub-optimal placement and misplacement according to the position of the catheter tip. The complications reported are catheter-related hemorrhage, intracranial infection and hydrocephalus. There were 105 cases enrolled, with 72 patients in the iUS-guided group having 131 catheters inserted and 33 patients in the group where ultrasound was not used with a total of 59 catheters. 116 (88.55%) were optimally placed, 12 (9.16%) sub-optimal and 3 (2.29%) misplaced in the iUS-guided group, while 25 (42.37%) were in optimally placed, 30 (50.85%) sub-optimal and 4(6.78%) misplaced in the control group. Accuracy of placement was highly significantly improved using ultrasound (P < 0.001). The operation time and the average catheterized time were longer in the iUS-guided group (P < 0.05), but the complication rates were no different between the groups. The mRS at three months was not significantly different between the two groups. Using iUS to place EVD catheters in patients with severe IVH is a safe technique delivering more accurate catheter placement without increasing the complication rate compared with freehand placement.

## Introduction

Compared with intracranial hemorrhage (ICH) alone, the mortality of ICH with intraventricular hemorrhage (IVH) is dramatically increased from 19.5% to 51.2%^1^. IVH is an independent risk factor for poor outcome^1,2^. External ventricular drainage (EVD) is most commonly used for cerebrospinal fluid (CSF) drainage, intracranial pressure (ICP) relief, and drug administration. However, there are many drawbacks to its use including slow drainage, frequent blockage of the catheter and increased risk of intracranial infection. Although EVD for chronic hydrocephalus is regarded as appropriate^3^, there is a lack of consensus for its use in acute IVH.

There is a very high rate of sub-optimal placement or misplacement of the EVD catheter by hand, in both hydrocephalus and with normal ventricles. Sub-optimal placement and misplacement was found in up to 45% (76/170) in ventriculoperitoneal (VP) shunt insertion without imaging guidance^4^. The main reasons include difficulty identifying the key anatomical markers, midline shift, neurosurgical inexperience and choosing the wrong length catheter^5^. Therefore, stereo-tactically guided EVD is recommended where there are abnormal ventricles, such as slit ventricles and complex hydrocephalus^6–8^. Intraoperative ultrasound (iUS) is widely used in neurosurgery, including brain tumor removal^9,10^, VP shunt^11–13^, and the drainage of brain abscess^14^. It has also been reported for use when there is midline shift, narrow ventricles and difficult anatomy^15^. To our knowledge, there have been few reports of iUS-guided management of IVH.

## Patients and methods

### Patients and Ethics

Patients who had iUS-guided EVD for severe IVH (the iUS-guided group) confirmed with computed tomography (CT) were enrolled retrospectively from January 2016 to July 2022 in our hospital and compared with the freehand group (the control group). Patients with a high risk of rebleeding due to coagulation dysfunction were excluded. The duration of drainage was no more than 2 weeks until either the ventricular obstruction was relieved, or more than 80% of the hematoma removed or the patient's condition worsened.

The trial was approved by the Ethics Committee of Taizhou First People’s Hospital in China (No.2022-KY119-01), adherence to the Declaration of Helsinki. The patient who was 18 year old or below, informed content must be obtained from a legal guardian, while above 18, informed consent must be obtained from the individual participant, or from a family if the patient was unconscious. The patients’ characteristics and 3 month outcome using the Modified Rankin Scale (mRS) were compared between the two groups.

### The iUS equipment

**Figure 1:**
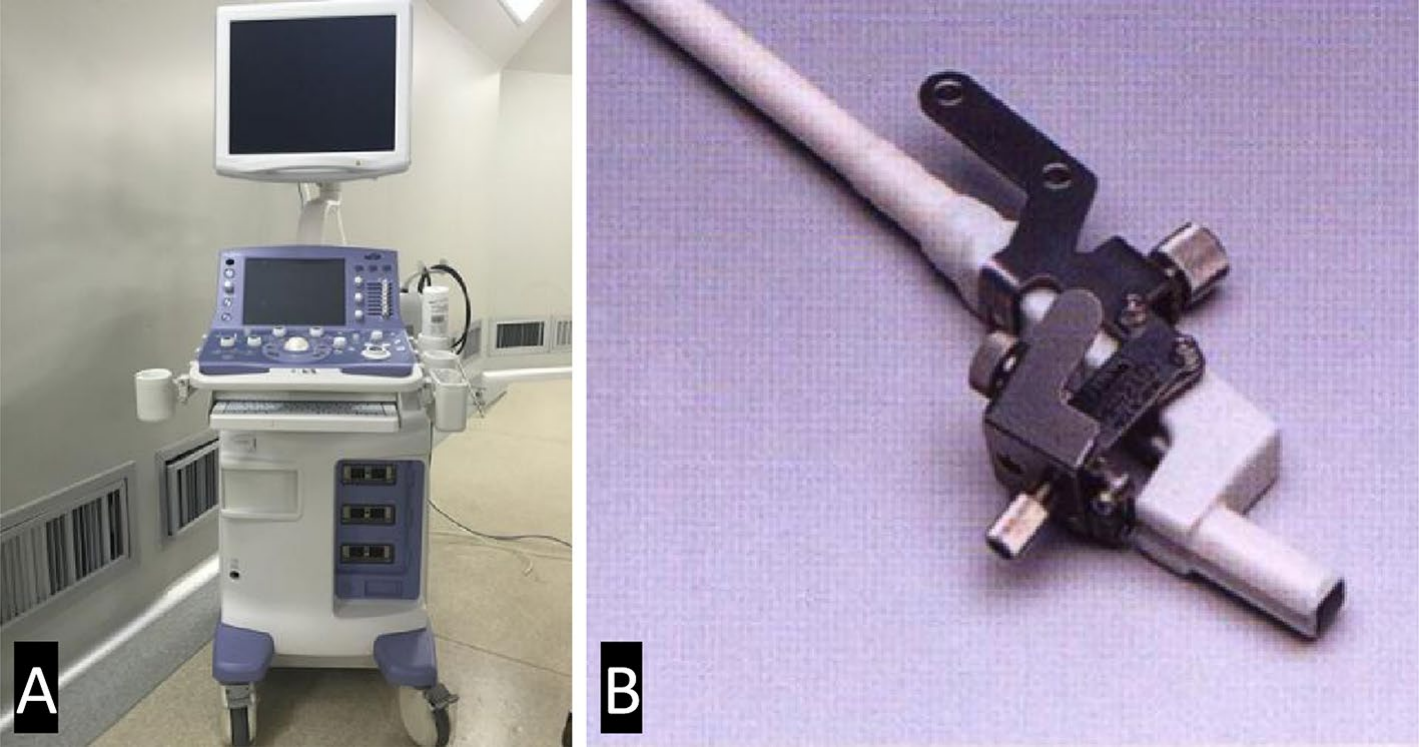
Intraoperative ultrasound equipment (**A**) and intraoperative electronic phased array probe for neurosurgical puncture (**B**).

The iUS (Aloka a7, Hitachi, Japan, Fig. 1A) was used with an intraoperative electronic phased array probe for neurosurgical puncture (UST-52114P, Hitachi, Japan, Fig. 1B).

### Puncture technique

**Figure 2:**
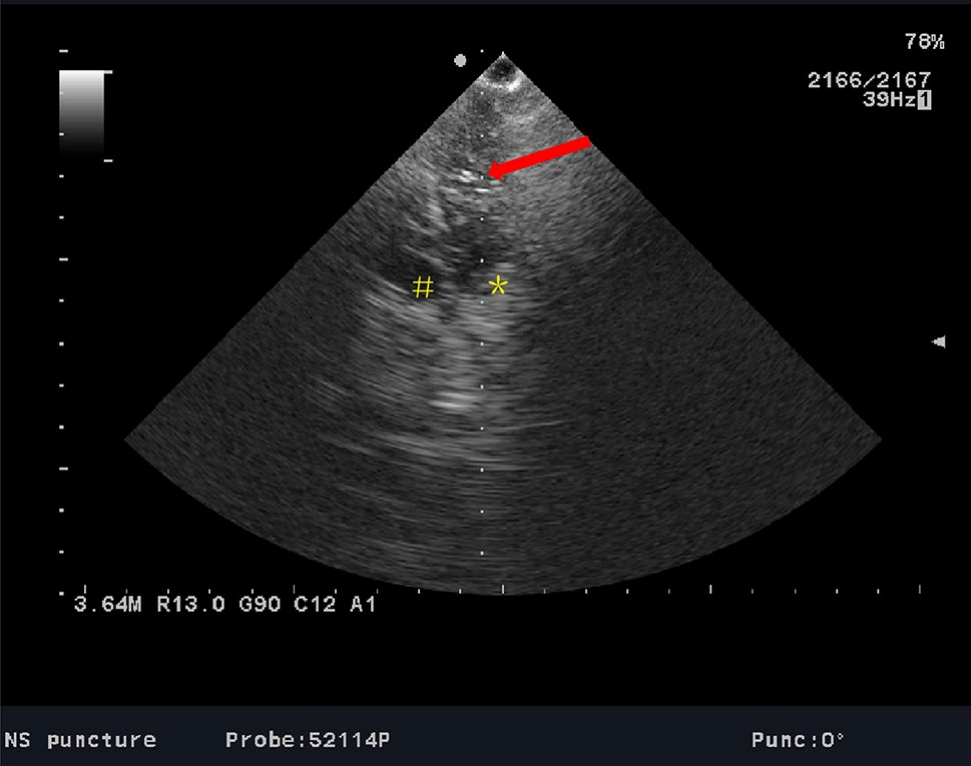
The image shown on the screen of iUS.The virtual puncture line (The dotted line shown by the red arrow), * the right ventricle, # the left ventricle.

All the EVDs in both groups were performed by the senior neurosurgeons with more than 5 years. Cranial puncture was through Kocher’s point, the most commonly point used for EVD in neurosurgery^16^. A burr hole was 3 cm lateral to midline and 11 cm superior and posterior from the nasion, or along the midpupillary line and 1 to 2 cm anterior to the coronal suture. In the control group as aspiration of blood stained CSF was rarely obtained and there was no access to intraoperative CT, it was necessary to aspirate with an empty needle or to inject no more than 3 ml of 0.9% saline and then to aspirate in order to confirm the accuracy of the catheter placement. In the iUS-guided group, the skull burr hole was enlarged to 1.2 cm, suitable for the probe and the EVD catheter (Fig. 1B). The location of the lateral ventricle was identified using iUS, and then, the dimension of EVD catheter was determined according to the virtual trajectory with the probe of iUS kept in a stable position (Fig. 2).

The EVD catheter (Codman, Johnson & Johnson Co. USA or Sewoon Medical Co. Ltd. Korea) was placed carefully along the virtual trajectory shown by the iUS (Supplementary Video). Without a hole at the tip of EVD catheter, the length of the catheter had to be inserted 3 mm more than that shown by the iUS to ensure effective EVD drainage. Even if the catheter was placed properly, there may be little or no hemorrhagic CSF outflow, which makes the optimal placement of the EVD catheter difficult to be judged correctly during the operation where there is no intraoperative CT scan. Given that the catheter may be in place for a long time in patients with severe IVH, it was carefully placed more than 5 cm under the gap aponeurosis of the scalp before bringing out of the scalp so as to reduce the risk of intracranial infection.

### Determining the accuracy and safety of EVD

The accuracy was determined based on the tip position of the EVD catheter using postoperative CT^5^. The following criteria were used: Optimal placement (Grade 1): the tip of the EVD catheter was placed at the foramen of Monroe or the ipsilateral frontal part of the lateral ventricle. Sub-optimal placement (Grade 2): placed in other parts of the lateral ventricle, the third ventricle, or the contralateral ventricle. Misplacement (Grade 3): placed in the cerebral parenchyma. Two complications were recorded, catheter-related hemorrhage and intracranial infection.

### Statistical analysis

We used mean ± SD for normally distributed continuous variables, or medians for abnormally distributed continuous variables. To conduct a comparison between quantitative variables with normally distribution, we used the independent t-test for the comparison between two means. For the comparison of quantitative variables with abnormally distribution, we applied Mann–Whitney U test. Categorical variables were expressed as counts with percentages using the chi-square test or continuity correction test. The data was analyzed with the SPSS software (SPSS Inc. Chicago, IL, USA, version 21.0). All P-values reported are two-sided, and the statistical significance was set at P < 0.05.

## Results

From January 2016 to July 2022, there were 105 IVH patients recruited to the study who had EVD performed in our hospital. Seventy-two cases with severe IVH received iUS-guided EVD for the purpose of draining hemorrhagic CSF and alleviating ICP, and 33 Cases by who had EVD placement done without ultrasound. The characteristics of the patients are shown in Table 1. Compared with the characteristics of the two groups, there was only a statistically significant difference in age (P < 0.001). IVH secondary to ICH was the most common scenario in both the iUS-guided group and the control group (80.56% vs. 81.82%), and most of them were severe IVH with Glasgow Coma Score (GCS) less than 9 scores at baseline (56 vs. 26).

**Table 1:** The characteristics of the patients in the iUS-guided group and the control group.

Variable	iUS-guided (n = 72)	By hand (n = 33)	P-value
Sex, n (%)			
Males	52 (72.22)	23 (69.70)	0.790
Female	20(27.78)	10(30.30)	
Age, y, Median(range)	62 (28–86)	57 (38–88)	0.000
GCS score, n (%)			
3–5	25 (34.72)	13 (39.39)	0.905
6–9	31 (43.06)	13 (39.39)	
10–12	7 (9.72)	4 (12.13)	
13–15	9 (12.50)	3 (9.09)	
Drug administrated coagulation-related, n (%)			
Aspirin	17 (23.61)	6 (18.18)	0.281
Rivaroxaban	0 (0)	1 (3.03)	
Catheter-related hemorrhage	5 (6.94)	2 (6.06)	
Causes of IVH, n (%)			
IVH secondary to ICH	58 (80.56)	27 (81.82)	0.610
IVH	8 (11.11)	3 (9.09)	
Aneurysm	3 (4.17)	1 (3.03)	
Moyamoya	2 (2.78)	0(0)	
AVM	1 (1.39)	1 (3.03)	
Aspirin after carotid artery stent implantation	0(0)	1 (3.03)	

Compared with the characteristics of the two groups, there was a statistically significant difference in age (P < 0.001). IVH secondary to ICH was the main type in both group (58 vs.27), and most of the patients were severe IVH with GCS less than 9 scores at inpatient (56 vs. 26).

**Figure 3:**
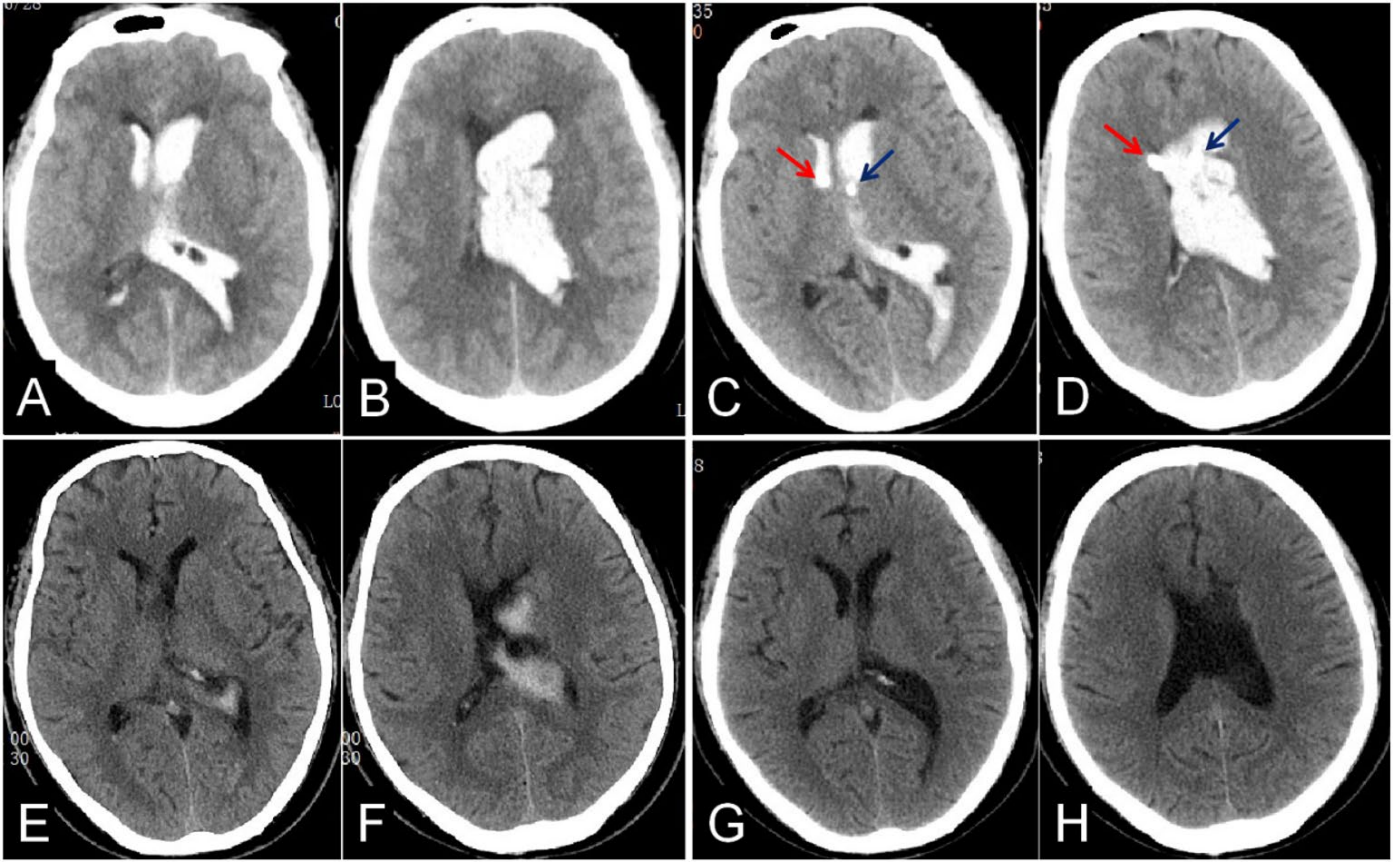
The optimal placement of EVD catheter was shown by the CT scan. IVH was shown at 3 h pre-operation (**A,B**); The locations of the EVD catheters were shown at 1 d post-operation with iUS-guided (the left catheter shown by the red arrow, the right catheter shown by the blue one, (**C,D**); There was about 80% IVH removal, and the catheter was removed at 2 weeks after EVD (**E,F**); And there was no hydrocephalus at 1 m follow-up (**G,H**).

**Figure 4:**
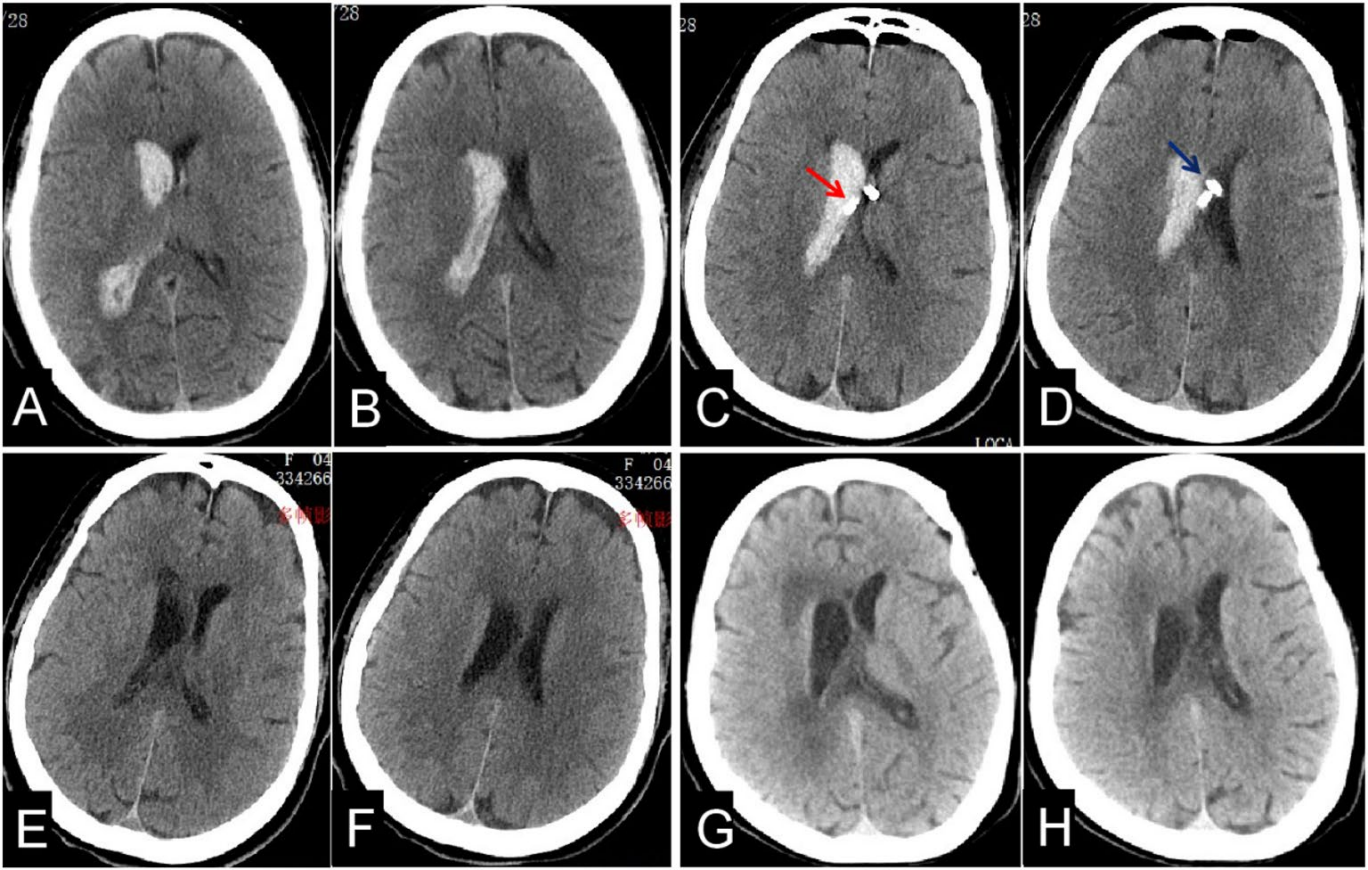
The sub-optimal placement of EVD catheter was shown by the CT scan. IVH was shown at 3 h pre-operation (**A,B**); Both EVD catheters were shown in the contralateral ventricle at 1 d post-operation by freehand (the left catheter shown by the red arrow and the right one shown by the blue one, (**C,D**); The catheters were removed and IVH was completely cleared at 2 w after EVD (**E,F**); The right ventricle was enlarged at 1 m follow-up (**G,H**).

**Figure 5:**
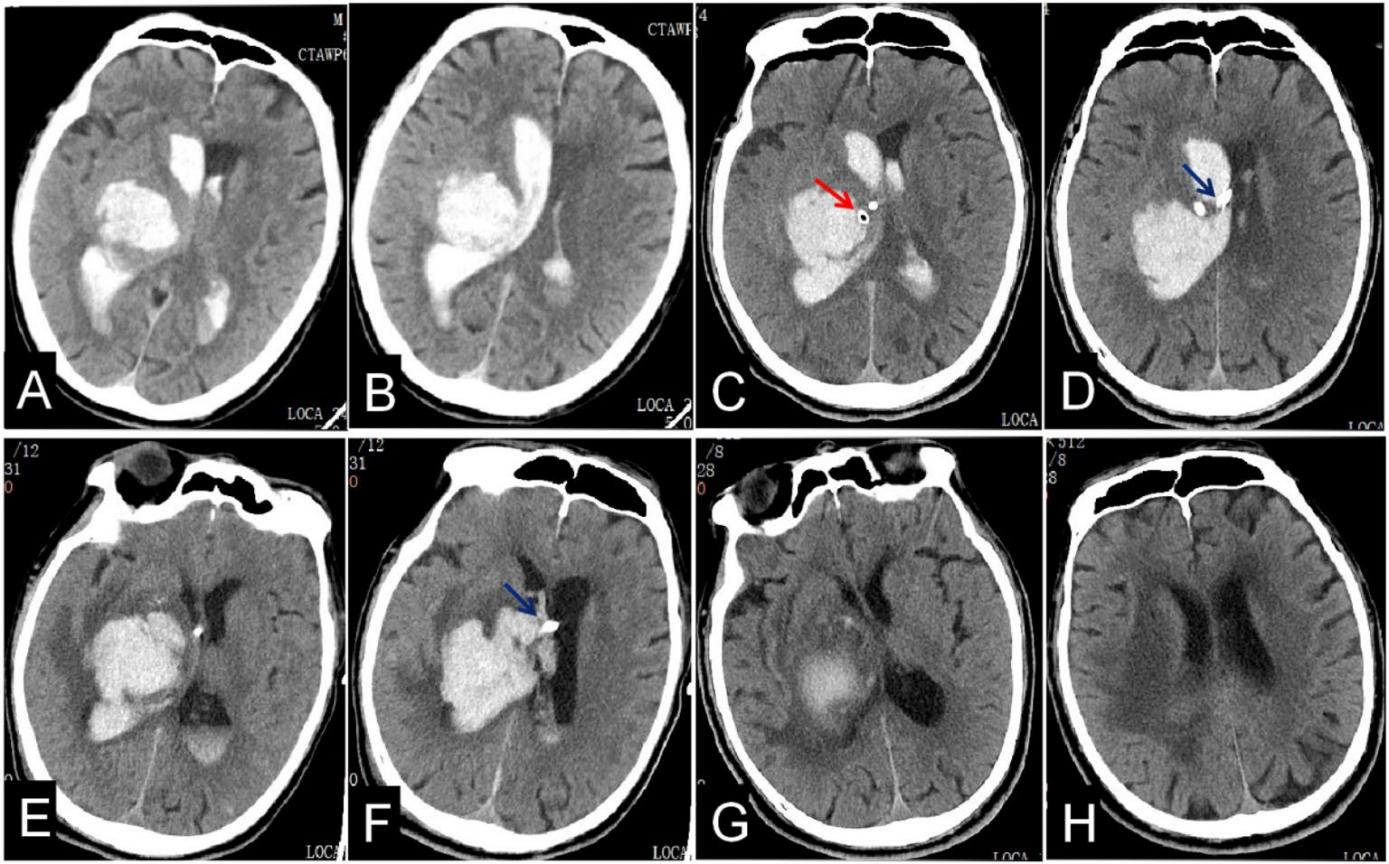
The misplacement of EVD catheter was shown by the CT scan. IVH was shown at 5 h pre-operation (**A,B**); The locations of the EVD catheters were shown at 1 d post-operation by freehand (the left catheter shown by the blue arrow in the contralateral ventricle, and the right catheter shown by the red one in the cerebral parenchyma, (**C,D**); The right catheter was removed next day after the CT scan post-operation (**E,F**); There was no hydrocephalus shown at 1 m follow-up, at the same time, uncomplete clearance of ICH (**G,H**).

Table 2, gives the operation and catheterization times which were significantly longer in the iUS-guided group than those in the control group (P < 0.05). The rates of catheter related hemorrhage, intracranial infection and hydrocephalus were no differences between the two groups. There was a trend towards shorter catheter removal times in the iUS-guided group, however, this did not reach statistical significance (P = 0.071). Interestingly, the accuracy of the EVD catheter placement was markedly higher in the iUS-guided group than that of the control group (88.55% vs. 42.37%, P < 0.001), which resulted in a better drainage (Fig. 3). In the control group, sub-optimal placement was considerably higher than in the ultrasound group (50.85%, Fig. 4). Misplacement into the brain parenchyma only occurred in three patients in the ultrasound group and four patients in the control group (Fig. 5). The drainage time was more than two weeks in one case in the iUS-guided group and three in the control group due to catheter-related intracranial infection requiring drainage of the infected CSF. There was no significant difference in the mRS at 3 m follow-up between the two groups (P = 0.382).

**Table 2:** The results in the iUS-guided group when compared with the control group.

Variable	iUS-guided (n = 72)	By hand (n = 33)	P-value
Time, mean ± SD			
Onset to operation (h)	6.13 ± 4.32	9.14 ± 20.53	0.236
Operation (min)	70.21 ± 15.98	59.88 ± 16.44	0.003
Average of catheter placed (min)	21.72 ± 3.10	17.54 ± 3.98	0.000
Comorbidities, n (%)			
Catheter-related hemorrhage^a^	14 (10.69)	9 (15.25)	0.433
Intracranial infection	8 (11.11)	5 (15.15)	0.791
Hydrocephalus	5 (6.94)	3 (9.09)	1.000
Intraventricular urokinase injection	22 (30.56)	6 (18.18)	0.183
Drainage time (d), n (%)^b^			
≤ 3	16 (22.22)	6 (18.18)	0.071
4–10	26 (36.11)	7 (21.21)	
11 ~ 14	29 (40.28)	17 (51.52)	
> 14	1 (1.39)	3 (9.09)	
Catheter position accuracy, n (%)^c^			
Grade 1	116 (88.55)	25 (42.37)	0.000
Grade 2	12 (9.16)	30 (50.85)	
Grade 3	3 (2.29)	4 (6.78)	
Outcome at 3 months by mRS, n (%)^d^			
0	3 (4.35)	1 (3.22)	
1	7 (10.14)	4 (12.90)	
2	9 (13.04)	1 (3.22)	
3	6 (8.70)	2 (6.45)	
4	8 (11.59)	5 (16.13)	
5	10 (14.49)	4 (12.90)	
6	26 (37.69)	14 (45.16)	
Favorable outcome (mRS 0–2), n (%)	19 (27.54)	6 (19.35)	0.382
Unfavorable outcome(mRS 3–6), n (%)	50 (72.46)	25(80.65)	

^a^There were 14 cases of catheter-related hemorrhage in the iUS-guided group having 131 catheters inserted, while 9 in the control group with 59 ones.

^b^There was a trend towards shorter catheter removal times in the iUS-guided group, however, this did not reach statistical significance (P = 0.071).

^c^There were 131 catheters inserted in 72 patients in the iUS-guided group, and 59 in 33 cases in the control group.

^d^There were 69 patients evaluated mRS at 3 months in the iUS-guided group and 31 in the control group.

## Discussion

The treatment of severe IVH is a major challenge in neurosurgery^1–3^. When acute hydrocephalus is present radiologically or clinically, EVD is recommended in guidelines but with a low level of evidence^3^. The incidence of complications and misplacement of EVD is up to 40% when performed freehand^17^, showing the need for major improvements. The optimal placement is improved greatly with stereotaxy^18^ or iUS^19^. A total of 51 VP shunt catheters were placed by stereotaxy with an accuracy of 88%^20^, while only 44.1% performed accurately when done freehand in a recent review^7^. However, there are a great many EVDs performed by freehand in clinical practice, partly because of the time consuming preoperative CT scan and registration for the stereotactic operation. It therefore tends to be used only where there is difficult anatomy such as slit ventricle or other complex anatomies^6^. Compared with freehand, the precision of EVD catheter placement using neuronavigation-guidance or XperCT-guidance is significantly improved from 69.2% to 90.2%^21^. But all these techniques require CT image acquisition and subsequent planning which can delay catheter insertion which may be critical in a severely ill patient. iUS-guided EVD may provide a quicker and easier way of delivering accurate catheter placement in these very sick patients.

The use of iUS was first reported in 1981 for the placement of a ventricular shunt^22^.With a high resolution and convenience of iUS, there is currently an iUS probe for puncture and real-time intraoperative use that provide high resolution and convenience. Compared with stereotactics-guided placement there are advantages including cost, real-time imaging and no need for preoperative CT image registration. Recently, iUS has been widely used in EVD for hydrocephalus^23^, VP shunt^11–13,24^ and EVD placement in normal ventricles^25,26^. But there is a lack of evidence for the iUS-guided EVD in IVH.

In this study, we found that the optimal placement of the EVD catheter was 88.55% (116/131) using iUS-guided in IVH, which was significantly better than those patients managed without ultrasound and similar to that achieved in previous reports using stereotactics-guided^20^ or neuronavigation-guided placement^21^. There were 3 cases of misplacement in the iUS-guided group and 4 cases in the control group, which may be caused by inexperience of the neurosurgeons in using of iUS, lateral ventricle compression and midline shift. Due to the massive IVH, bilateral placement was performed in most of our cases (59/72 vs. 26/33) to increase the chances of unobstructed drainage on at least one side. The virtual trajectory is shown on the iUS, so the optimal placement can be adjusted before the puncture. EVD placement has also been reported using a flexible endoscope for IVH. It resulted in significant reduction in hydrocephalus but no improvement in one year outcomes^27^. Another small study with 25 IVH cases used neuroendoscopy to remove the haemorrhage, which could reduce length of time needed in ICU and with some possible improvement in neurological outcome^28^, however the sample was too small and not randomised. Neuroendoscopy for the management of IVH requires a randomised trial yet.

Currently, EVD is recommended for IVH in the American Heart Association guideline^3^. Data suggests that, there were approximately 500,000 ventriculostomies performed in the United States from 1988 to 2010^29^, so even a little progress in the optimal placement of the EVD catheter could benefit a large number of the IVH patients. We believe that the placement of the EVD with iUS-guidance is more convenient than stereotactics-guided and neuronavigation-guided placement.

Another purpose of the EVD is intraventricular injection of thrombolytics in IVH, so optimal placement is essential. A study found that the poor outcome was only 31.8% in IVH with urokinase administered, compared to 66.7% with EVD alone^30^. A systematic review has found that EVD is still a standard treatment in IVH with hydrocephalus, and intraventricular injection of thrombolytics commonly used for rapid hemorrhagic removal in IVH^31^. The clinical trial, CLEAR III, found that intraventricular injection of recombinant tissue plasminogen activator (rt-PA) was safe with no related symptomatic hemorrhage in IVH^32^.

A previous study showed that the incidence of intracranial infections associated with the EVD placement was 18%^33^. In our group, there were only 9.72% (7/72) in the iUS-guided group and 9.09% (3/33) in the control group, perhaps partly because the drainage time of IVH was less than 2 weeks and the catheter was tunneled more than 5 cm under the gap aponeurosis of the scalp before bringing out of the scalp for those patients needing a longer drainage time.

The accuracy of the EVD catheter placement was markedly higher with iUS-guidance compared with that by freehand, however, there was no significant improvement in the outcomes at three months, this may be the result of a relatively small number of patients in the two groups. The statistical results of patients’ characteristics showed that there was a difference in age between the two groups. There were no inclusion criteria of a selective difference in age in this retrospective study. The median age of patients was older in the iUS-guided group than that in the control group (P < 0.001), which might be more severe IVH due to brain atrophy or other reasons that leaded neurosurgeons to choose the accuracy of the iUS. In addition, there may be a certain chance of the difference in age between the two groups. Although the difference of age might affect outcomes, it had no effect on the accuracy of catheter placement with iUS-guided.

## Limitations

The study was relatively small and the ultrasound managed group and the control group were not randomised, which is likely to have introduced some bias into the results. Patients were recruited from a single centre and therefore further studies, with randomised groups are needed to be confident that our results are applicable in other settings, both within China and internationally.

## Conclusion

The optimal placement of the EVD catheter is significantly improved with iUS-guided compared with that performed freehand, and is equal to that of previously reported stereotaxy-guided or neuronavigation-guided insertion. We believe it saves time without increasing risks of catheter-related hemorrhage and intracranial infection.

## Supplementary Information

Supplementary Video 1.

## Data Availability

The data will be provided by the corresponding author when required.

## Abbreviations

ICH: Intracranial hemorrhage
IVH: Intraventricular hemorrhage
EVD: External ventricular drainage
CSF: Cerebrospinal fluid
ICP: Intracranial pressure
VP: Ventriculoperitoneal
iUS: Intraoperative ultrasound
CT: Computed tomography
AVM: Arteriovenous malformation
mRS: Modified Rankin Scale
rt-PA: Recombinant tissue plasminogen activator
